# Cheek Injury: A Rare Cause of Pneumomediastinum

**DOI:** 10.7759/cureus.95834

**Published:** 2025-10-31

**Authors:** Wim Van Cleemput, Thomas Vanderveren, David Ruttens

**Affiliations:** 1 Pulmonary Medicine, University of Antwerp (UAntwerp), Antwerp, BEL; 2 Pulmonary Medicine, Ziekenhuis Oost-Limburg (ZOL) Genk, Genk, BEL

**Keywords:** causes of pneumomediastinum, cheek injury, pneumomediastinum (pm), secondary pneumomediastinum, self-inflicted trauma to the buccal mucosa

## Abstract

Pneumomediastinum is a rare condition characterized by the presence of air within the mediastinal space. It may occur spontaneously, often due to raised intrathoracic pressure, or secondarily from trauma, infection, or iatrogenic causes. Self-inflicted intraoral trauma is an exceptionally rare etiology but should be considered in recurrent or unexplained cases.

A young woman with a history of depression and a chronic motor tic leading to habitual cheek biting presented with facial swelling and extensive subcutaneous emphysema after biting her right buccal mucosa. Imaging demonstrated cervicofacial emphysema extending into the deep neck spaces and upper mediastinum, consistent with pneumomediastinum. No evidence of alveolar rupture, infection, or other intrathoracic pathologies was found. A similar episode had occurred six weeks earlier, likewise attributed to cheek trauma. Apart from a large traumatic ulcer of the right buccal mucosa, no other cause, such as asthma, Valsalva-like activities, trauma, or dental procedures, was identified, making the self-inflicted injury the most likely etiology. She was observed in intensive care, remained stable, and was discharged with a nocturnal mouthguard and outpatient follow-up.

This case illustrates an unusual mechanism of pneumomediastinum through direct mucosal injury and air tracking along fascial planes. Recurrent episodes highlight the behavioral component, warranting both medical and psychosocial attention. Conservative management proved effective, with prevention focusing on behavioral modification and protective measures.

Unusual causes of pneumomediastinum, including self-inflicted oral trauma, must be considered. Careful history-taking, imaging, and preventive strategies enable safe management and reduce recurrence.

## Introduction

Pneumomediastinum, also referred to as mediastinal emphysema, is defined as the presence of air within the mediastinal space [1]. It usually results from the leakage of air from the lungs, airways, or esophagus, which then migrates into the mediastinum. Although often self-limiting, pneumomediastinum can occasionally compromise vital thoracic structures such as the heart, tracheobronchial tree, and great vessels.

The condition is broadly classified into two forms: spontaneous (primary), occurring in otherwise healthy individuals without an identifiable cause, and secondary, arising from an underlying pathology such as trauma, infection, or iatrogenic injury. The most common underlying mechanism involves alveolar rupture with subsequent air dissection along the bronchovascular sheaths [2].

Here, we describe a rare case of secondary pneumomediastinum caused by self-inflicted orofacial trauma. While subcutaneous emphysema following oral or maxillofacial injury has been described [1], extension through the cervical fascial planes into the mediastinum is uncommon and only rarely reported [2]. This case highlights an unusual pathway for air dissection and underscores the relevance of behavioral factors in its recurrence.

## Case presentation

**Figure 1:**
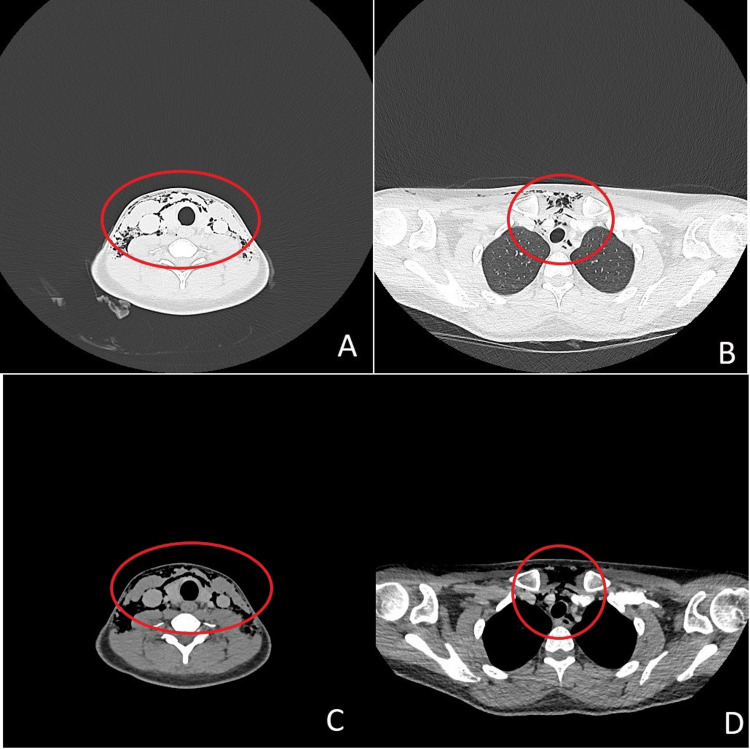
CT scan of the first episode showing subcutaneous emphysema in the neck and pneumomediastinum (A) Neck imaging in lung window, (B) thoracic imaging in lung window, (C) neck imaging in soft tissue window, and (D) thoracic imaging in soft tissue window CT: computed tomography

A 26-year-old woman with a history of depression, a chronic motor tic causing habitual cheek biting, and active tobacco use presented to the emergency department with extensive subcutaneous emphysema. She reported awakening the morning after an evening of social activity with progressive neck swelling. On examination, crepitus was palpable over the neck and lower face, without respiratory distress or cardiovascular compromise. A computed tomography (CT) imaging of the neck and thorax (Figure 1) demonstrated cervicofacial emphysema extending into the deep cervical spaces and upper mediastinum, consistent with pneumomediastinum. A traumatic ulcer on the right buccal mucosa was noted; no other precipitating factor was identified. The exact mechanism of pneumomediastinum in this patient remained unclear. No infection was identified, but empirical amoxicillin-clavulanate was started. She was managed conservatively, with close observation, and the pneumomediastinum resolved spontaneously. The patient remained in good condition and was discharged home after three days.

Six weeks later, she was readmitted with acute facial swelling and a sensation of subcutaneous air following an involuntary bite to the right buccal mucosa the day before. She reported minimal shortness of breath and denied fever or recent illness. She was alert and in no acute distress. Vital signs were as follows: heart rate of 94 beats per minute, blood pressure of 113/68 mmHg, respiratory rate of 14 breaths per minute, temperature of 36.7°C, and oxygen saturation of 95% on room air. Marked subcutaneous emphysema involved the face, periorbital region (predominantly on the right), and thorax. Cardiac examination revealed tachycardia with regular rhythm and normal heart sounds; pulmonary auscultation showed bilateral vesicular breath sounds with fine crepitations in the right apical region.

**Figure 2:**
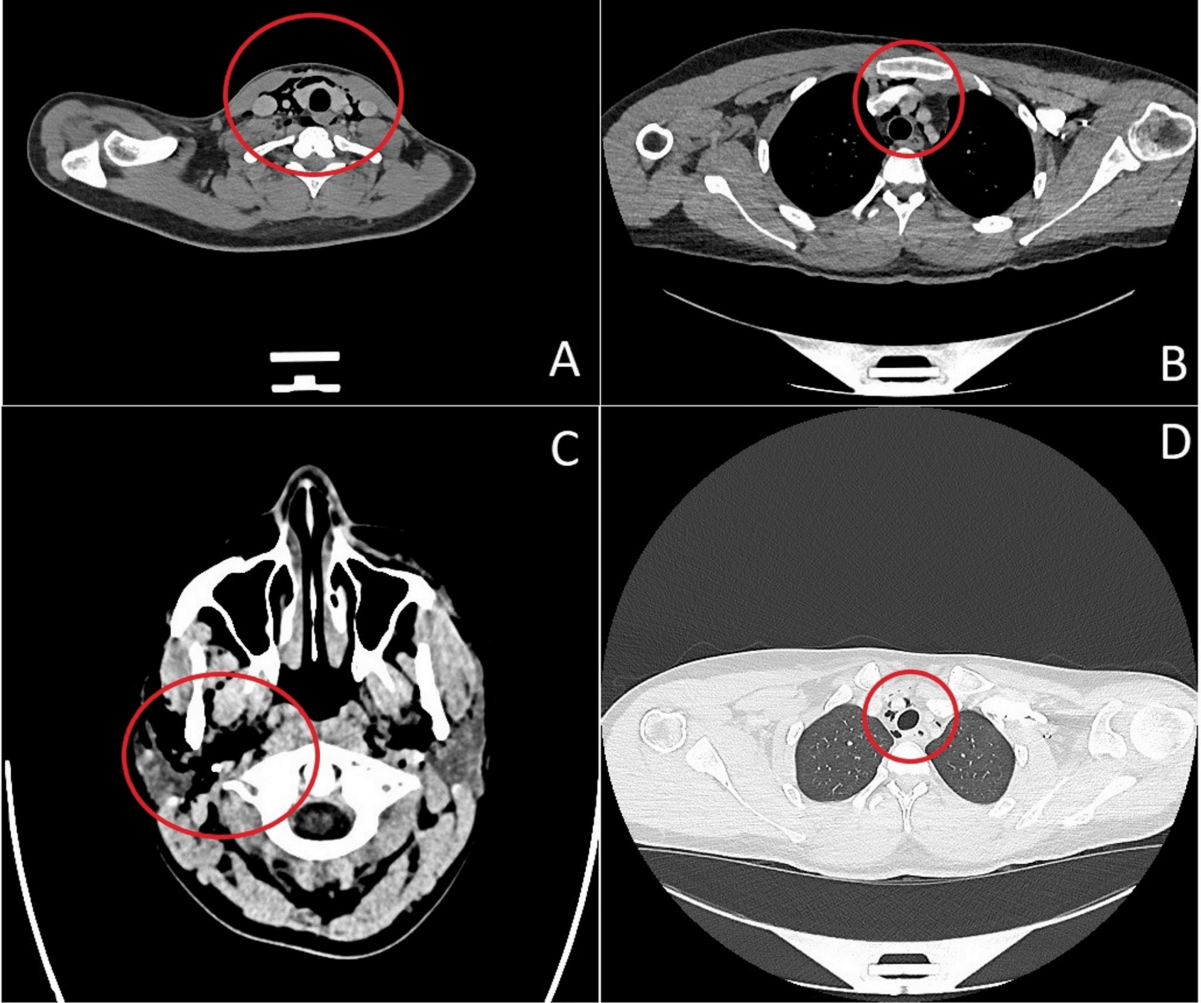
CT scan at second presentation at the emergency care (A) Upper thoracic imaging in soft tissue window, (B) thoracic imaging in soft tissue window, (C) CT scan of the skull in soft tissue window, and (D) thoracic imaging in lung window CT: computed tomography

Repeat CT imaging of the head, neck, and thorax (Figure 2) confirmed extensive emphysema in the right periorbital and buccal regions, as well as in the deep cervical spaces bilaterally, including the retropharyngeal area, with extension into the upper mediastinum. No abscess or intracranial pathology was observed.

**Figure 3:**
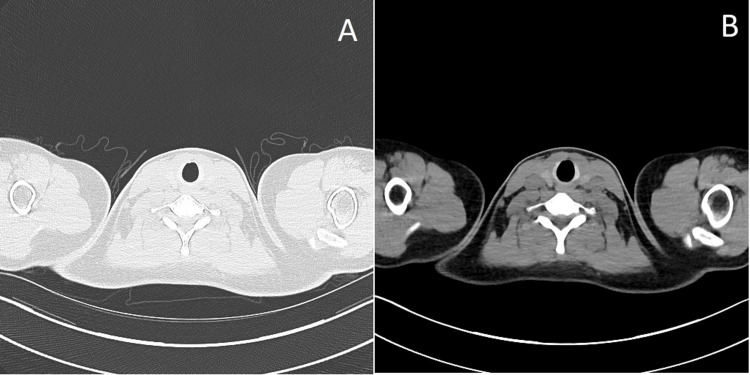
Control CT scan showing no remaining subcutaneous air (A) Thoracal imaging in lung window and (B) thoracal imaging in soft tissue window CT: computed tomography

The patient was admitted to the intensive care unit for close monitoring. Her clinical course remained uncomplicated, allowing transfer to the general ward. Examination by an ear, nose, and throat specialist confirmed a large traumatic ulcer on the right buccal mucosa. A nocturnal mouthguard was fitted by the maxillofacial team. Pre-discharge imaging demonstrated the regression of the pneumomediastinum, suggesting no active air leak. Outpatient follow-up, including a repeat CT of the thorax seven weeks later, showed the complete resolution of the prior pneumomediastinum (Figure 3).

The recurrent episodes, in the absence of other common causes such as asthma, physical exertion, trauma, or asphyxiation, indicated self-inflicted oral trauma as the most likely etiology.

## Discussion

Pneumomediastinum is an uncommon condition that can arise spontaneously or secondarily. Clinically, it often presents with subcutaneous emphysema or palpable crepitus, as seen in our patient. CT allowed a rapid diagnosis of pneumomediastinum in this case. Pneumomediastinum should be considered in all patients presenting with subcutaneous emphysema and should be investigated. Although typically self-limiting, pneumomediastinum can occasionally lead to serious complications such as the compression of mediastinal structures or mediastinitis [3]. A fast workup is consequently paramount.

Secondary pneumomediastinum most commonly originates from the lower respiratory tract. However, rare cranial sources have been described. Intraoral defects, including traumatic cheek bites, can serve as entry points for air, which may pass through the buccinator muscle into adjacent soft tissues and extend via the parapharyngeal and retropharyngeal spaces into the mediastinum. This pathway is anatomically plausible and has been reported in the literature following both traumatic and iatrogenic oral injuries [2,4-7].

Our patient experienced recurrent episodes of pneumomediastinum following self-inflicted cheek biting, a mechanism that has been sparsely reported. The presence of a chronic motor tic likely predisposed her to repeated mucosal trauma, emphasizing the behavioral component in this case. The recognition of such unconventional triggers is crucial, as even minor intraoral injuries can result in extensive subcutaneous emphysema and mediastinal air tracking [4-7].

The management of pneumomediastinum is primarily conservative in stable patients, including observation, supplemental oxygen when indicated, and the treatment of underlying causes. In this case, preventive measures, specifically the fitting of a nocturnal mouthguard, were implemented to reduce the risk of recurrence. The favorable clinical course and complete resolution on follow-up imaging demonstrate that conservative management, combined with behavioral interventions, is effective in such cases.

This report underscores the importance of thorough history-taking, careful physical examination, and early imaging in patients presenting with subcutaneous emphysema. The awareness of rare etiologies, such as self-inflicted oral trauma, allows timely diagnosis and tailored preventive strategies. In recurrent or unexplained pneumomediastinum, oral self-injury should be considered as a potential source, particularly in patients with psychiatric or neurological conditions predisposing to habitual trauma. A multidisciplinary approach, combining behavioral support, protective devices, and close clinical follow-up, is essential to prevent recurrence and optimize patient outcomes.

## Conclusions

Self-inflicted trauma to the buccal mucosa is a rare but clinically significant cause of pneumomediastinum. This case highlights that even minor intraoral injuries can lead to extensive cervicofacial emphysema and mediastinal air tracking. Recurrent episodes, as seen in a patient with a chronic motor tic, underscore the need for a multidisciplinary approach, including behavioral support and mechanical preventive measures such as a nocturnal mouthguard. Clinicians should maintain awareness of atypical etiologies, including self-inflicted oral trauma, to ensure timely diagnosis and implement preventive strategies.
